# Estimation of lead time and overdiagnosis in breast cancer screening

**DOI:** 10.1038/sj.bjc.6604762

**Published:** 2009-01-06

**Authors:** P C Gøtzsche, K J Jørgensen, J Mæhlen, P-H Zahl

**Affiliations:** 1 Norwegian Institute of Public Health, PO Box 4404 Nydalen, Oslo N-0403, Norway

**Sir,**

It is reported that 7 years after the start of screening in Sweden, the incidence of invasive breast cancer in the screened age group was 69% higher than expected (Duffy *et al*, 2008). After adjustment for a lead time of 2.4 years and for the increased use of hormone replacement therapy, they found 39% excess.

We have reservations about adjustments for lead time (Zahl *et al*, 2008). Correction for lead time should only be done for those cancers that would have been diagnosed at a later time in the absence of screening. But many screen-detected cancers would not have come to the women's attention in their remaining lifetime, if they had not attended screening and are by definition overdiagnosed. If these cancers are not excluded from the calculation of lead time, the lead-time distribution will be artificially right-skewed, and the average lead time will appear to be much longer than it really is. Duffy *et al*, (2008) did not exclude such cancers from the calculation of lead time, but counted all excess cancers detected in a randomised trial as advanced diagnoses, when some of them were in fact overdiagnosed cases. They have therefore overestimated the lead-time effect.

Duffy *et al* (2008) recognise that the detection of non-overdiagnosed cancers should give rise to a drop in incidence when the women leave the screening programme. Quantifying such a compensatory drop is a less bias-prone method for adjusting the incidence increase for lead-time effects, as it makes no assumption about the average lead time. It is also very simple to use (see, e.g., Zahl *et al*, 2004).

We have done a systematic review of incidence trends in countries with organised mammography screening that presented data on breast cancer incidence for both screened and non-screened age groups for at least 7 years before screening and 7 years after screening had been fully implemented (Jørgensen and Gøtzsche, 2008). We were able to include data from United Kingdom, Canada, Australia, Sweden and Norway. We found that compensatory drops in the older age groups were small or absent, although major drops would have been expected if the lead time of 2.4 years were true. When we adjusted for these drops, we found 36% overdiagnosis of invasive breast cancer, in good agreement with the results of Duffy *et al* (2008), and 51% when we included carcinoma *in situ*.

Duffy *et al* (2008) mention that after prolonged follow-up of the Malmö randomised screening trial, an overdiagnosis of 7–8% was reported and they find this estimate considerably more plausible than their own estimate of 39%. However, they have overlooked that the former estimate is seriously flawed (Zahl *et al*, 2008). There was substantial opportunistic screening in the control group and after adjustment for this, the overdiagnosis estimate in the Malmö trial is 24% (Gøtzsche and Jørgensen, 2006).

It is asserted that after adjustments, overdiagnosis estimates will be smaller than many rates quoted in the past (Duffy *et al*, 2008). We disagree, as most ‘rates quoted in the past’ have been too small (Gøtzsche and Nielsen, 2006). On the basis of randomised trials, the overdiagnosis is 30% (Gøtzsche and Nielsen, 2006), and it becomes 44% if adjusted for opportunistic screening in the control groups (Jørgensen and Gøtzsche, 2008). The low rates in the past have been too low for the very reason that they have been based on flawed lead-time models (Jørgensen and Gøtzsche, 2008; Zahl *et al*, 2008). We believe the most reliable estimate for organised mammography screening is 51% (Jørgensen and Gøtzsche, 2008). This means that one in three breast cancers detected in a population offered organised mammography screening are overdiagnosed. Many women are therefore harmed substantially by screening, as practically all detected carcinoma *in situ* cases and invasive cancers are treated, at great physical and psychological costs.
